# Study on Recycling of Steel Slags Used as Coarse and Fine Aggregates in Induction Healing Asphalt Concretes

**DOI:** 10.3390/ma13040889

**Published:** 2020-02-17

**Authors:** Haiqin Xu, Shaopeng Wu, Hechuan Li, Yuechao Zhao, Yang Lv

**Affiliations:** State Key Laboratory of Silicate Materials for Architectures, Wuhan University of Technology, Wuhan 430070, China; xuhaiqin@whut.edu.cn (H.X.); wusp@whut.edu.cn (S.W.); zhaoyc@whut.edu.cn (Y.Z.); lvyang@whut.edu.cn (Y.L.)

**Keywords:** steel slag, recycling, induction heating, asphalt concrete

## Abstract

Steel slag, a by-product of steelmaking, imposes lots of negative impacts on the environment. For alleviating negative impacts, more and more experiments have been carried out to explore the application possibility of steel slag. The purpose of this study is to explore the feasibility of steel slag being applied in induction healing asphalt concretes to replace coarse and fine aggregate. Surface texture and pore sizes of steel slag were firstly tested, and then steel slag and basalt asphalt mixtures modified with steel fibers were prepared. Moisture susceptibility, dynamic stability, mechanical property, thermal property, induction heating speed, natural cooling speed and healing property of the asphalt mixtures were evaluated. Results showed that steel slags had more obvious holes in the surface while the surface area is much larger than that of basalt. Furthermore, steel fibers and steel slag both have dynamic stability, and steel fibers contribute to increased moisture resistance while steel slag is not. Steel slag asphalt concrete showed better mechanical property and better capacity to store heating. Steel slag asphalt mixtures had a similar heating speed to basalt asphalt mixtures but a significantly slower cooling rate. Finally, the induction healing test and CT scanning test demonstrated that steel slag asphalt mixtures had a similar healing ability to basalt asphalt mixtures. It can be concluded that steel slags have the potential to replace the natural aggregates to be applied in induction heating self-healing asphalt concretes.

## 1. Introduction

Asphalt concretes are widely used pavement materials all over the world. With excellent performances of low noise, comfortable driving and simple construction in AC grade asphalt pavements compared to concrete pavements, asphalt concretes were applied in more than 90% high-grade highways in China [1]. However, during the period of construction, hot-mix asphalt (HMA) concrete is used most usually, which contains over 90% of aggregates by weight [2]. Consequently, the construction of asphalt pavement has used up considerable natural aggregates. The pressure on provision of natural aggregates is forcing researchers to find substitutes of natural raw materials. Applying poorer natural raw materials [3] and recycling some large output wastes such as coal gangue [4], steel slag [5,6,7], reclaimed asphalt pavement (RAP) [8,9] etc. into asphalt pavement construction is a prospective way to relive the request of natural aggregates.

Steel slag is a main solid by-product in the production of iron and steel, which consists of up to about 13% of crude steel production [10,11]. With the rapid development of the Chinese economy, steel production is also gradually increasing, which certainly results in large amounts of steel slag accumulating. Nowadays, China is facing a problem that over one billion tons of steel slag accumulations need to be processed due to single application method previously, which has caused a series of issues to environment, such as arable lands occupation, water contamination and heavy metal pollution [12,13]. In recent years, researchers have tried to apply steel slag as an aggregate in road construction to alleviate the damage caused by steel slag accumulation [14]. Studies indicate that steel slag formed by crushing and proper process has a similar shape to the natural aggregates [15] and better mechanical properties [16], so it has the potential to become an aggregate for road building materials. The mechanical characteristics [17,18] and pavement performances [19] such as moisture stability [20,21], skid resistance [21,22] and crack resistance were improved with steel slag applied as a coarse aggregate in asphalt mixtures. However, steel slag replaced as a fine aggregate is not suggested owing to some poor performances and high cost factors. Due to some free lime (f-CaO) existing, its hydration leads to volume instability, so some studies suggested that the total expansion of steel slag should be less than 1% when used in an asphalt mixture [23,24]. Steel slag, as porous material, excesses asphalt absorption which leads to high cost on the one hand, and the existence of pores will make the steel slag possess good heating storage performance on the other hand. Steel slag possesses high density compared to natural aggregates, so the transportation also needs studying. Which kind of transportation is more suitable in economy and can reduce carbon emission should be studied further [25], and it must be admitted that long-term service of steel slag may cause some environmental impact though the use of steel slag in road engineering, which solves the environmental impact of a large amount of steel slag stacking. The accumulation of toxic metals released from BOF highly correlated with those in soil beneath construction site has been proven by Xie et al. [26].

Although new transportation roads are being built in large numbers in many countries, maintenance is a priority to other countries. Asphalt concretes are easily damaged by traffic loads, climates changing and UV lightings during service. Cracks are the most common damage, the expansion and extension rates of which are extremely fast once cracks are generated and then cracks can connect more easily to form larger cracks. Therefore, it is necessary to repair the asphalt pavements regularly to extend the service life. Generally, traditional repairing methods were applied after macroscopic distresses, which cannot fix the pavements well enough, but also lead to a large amount of waste of resources [27]. Induction heating, a new repairing method that possesses the ability to heal asphalt mixtures, is being studied by many researchers. The method was created according to the fact that asphalt can flow to heal itself if the outside temperature is above the glass transition temperature of asphalt [28]. The first induction heating asphalt concrete study was proposed by García et al. [29,30]. Many factors affect the self-healing properties of asphalt mixtures, including asphalt type, aggregate type, conductive addition type, heating temperature, and distance between surface of asphalt concrete and heating device [31,32,33]. The advantages of this method are high heating efficiency, being environmentally friendly, and the fact that the heated object does not beed to be in contact with the induction coil.

The purpose of this paper is to investigate the feasibility to apply steel slag into induction heating asphalt concretes to replace coarse and fine aggregates. Surface texture was observed by a Scanning Electron Microscope (SEM) and pore sizes were tested by Surface Area and Porosimetry Systems. Heating and cooling speed were recorded by an infrared camera. Thermal property was determined by a thermal constant analyzer. Healing property was evaluated based on the fracture–healing–fracture test.

## 2. Materials and Experiments

### 2.1. Materials

In this study, SBS-modified asphalt (Guochuang Hi-tech Material Co. Ltd, HuBei, China), basalt aggregate (Jinshan Songhe Aggregates Company, Yidu, China), limestone filler (Maliang Aggregates, Jinmen, China) were used to prepare asphalt concretes. The basic properties of asphalt and basalt aggregates are shown in Table 1 according to the standard test method in China (JTG E20-2011 and JTG E42-2005) and the specification was determined according to standard of JTG F40-2004.

In this research, Steel slag was used as both an aggregate and conductive material, and steel fibers were used for induction heating units. Steel slags were produced by Baotou Iron and Steel (Group) Co. Ltd, Inner Mongolia, China, and steel wool fibers were supplied by Jiangsu Golden Torch Metal products Co, Ltd, Jiangsu, China. The properties of steel fibers and steel slags were shown in Table 2, the specification of steel slag is collected from a Chinese standard GB/T 25824-2010.

### 2.2. Specimen Preparation

According to the Marshall design method, six kinds of AC-13 grade asphalt concretes were prepared in this study. BA means the asphalt concrete whose aggregates are all basalts; BA-6 is prepared by adding 6% steel fibers (by the volume of asphalt) to BA; SS means asphalt concrete whose aggregates were all steel slags; SS-2, SS-4, SS-6 are the asphalt concretes prepared by adding 2%, 4% and 6% steel fibers (by the volume of asphalt) to SS respectively.

**Figure 1:**
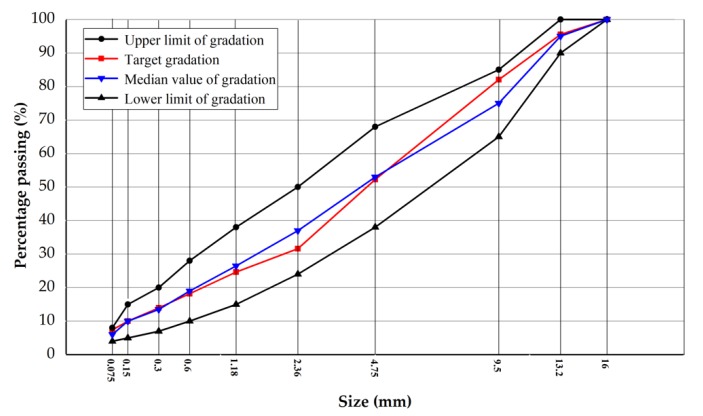
Gradation curves of the asphalt concretes.

In order to compare the performance better, the six kinds of asphalt concretes use the same gradation and the gradation curve is shown in Figure 1. The asphalt–aggregate ratio of the concretes without steel fibers is all 4.7% and it with steel fibers is 4.9%.

In this study, the heating and cooling test, SCB test and induction-healing test all adopted semi-circular Marshall specimens cut by standard Marshall specimens, the thickness of which is 25 mm, and at the bottom of which exists a groove of 20 mm in the middle of the radial direction. The groove is convenient for stress concentration and controlling cracks.

### 2.3. Moisture Susceptibility Test

Moisture damage can cause cracks on the asphalt film wrapped around the aggregate and the asphalt gradually falls off. In this paper, the freeze–thaw split test is used to evaluate the moisture susceptibility of different types of asphalt concretes. The specific steps of the test are carried out in accordance with T 0729-2000, JTG E20-2011. The test shows the ability of asphalt mixture to resist moisture damage by the freeze–thaw splitting tensile strength ratio. The larger the value, the better the moisture damage resistance.

### 2.4. High-Temperature Deformation Resistance Test

High temperature deformation resistance was tested by a wheel tracking device. The experiment shows the ability of asphalt concrete to resist rutting damage at high temperature with dynamic stability. The specimen used in this test is 300 × 300 × 50 mm in size, and the test was done under 0.7 ± 0.05 MPa repeated wheel loading at the temperature of 60 °C. The speed of wheel was chosen of 42 pass/min. After the test, the dynamic stability was obtained, and the larger the value, the better high temperature deformation resistance. This test was carried out according to the standard of T0719-2011, JTG E20-2011.

### 2.5. Semi-Circle Bending Fracture Test

In this paper, a semi-circular bending fracture test was used to study fracture energy and peak load of the asphalt concretes which are vital indicators for evaluating the crack resistance and the mechanical properties of different asphalt mixtures. Fracture energy is characterized as the area under the force-displacement curve between 0 and peak displacement. The displacement–load curve was obtained by a Universal Testing Machine (UTM-25) to break the samples into two pieces with the constant displacement of 5 mm/min.

### 2.6. Thermal Properties Test

A thermal properties test was used to determine the effect of steel fibers content and aggregate type on the thermal properties of asphalt concretes. Steel fibers are highly thermal conductive while steel slags are low thermal conductors due to many holes, which all have an impact on thermal properties. Thus, investigating thermal properties accurately is essential for the period of heating and cooling.

In this study, a Hot Disk Thermal Analyzer (TPS2500S, Hot Disk, Göteborg, Sweden) was used to test the thermal constant of asphalt concretes by transient flat heat source methods. A probe with a radius of 14.61 mm was chosen. Heating time was 40 s, and the test power was 80 mw. Test results include thermal conductivity, thermal diffusivity, and specific heat capacity.

$$SH=\frac{TC}{TD}$$

where *SH* is specific heat, MJ/(m^3^·K), *TC* is thermal conductivity, W/(m·K), *TD* is thermal diffusion, (mm^2^/s).

**Figure 2:**
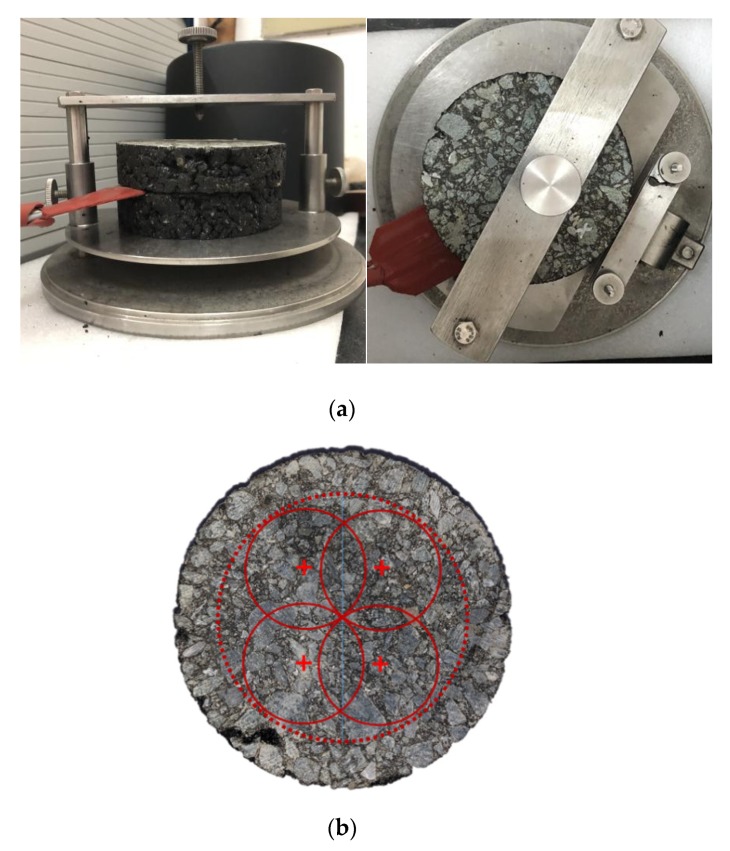
Test area of thermal properties test. (**a**) Test system; (**b**) Test area.

As shown in Figure 2a, the probe was placed in the middle of the pieces of the Marshall sample. However, the probe cannot cover most of the aggregate and mortar areas, which leads to the test results being unrepresentative. Actions should be done to reduce data skews. Four areas were chosen as shown in Figure 2b, and three tests were done in every area. The thermal constants of each sample were obtained by calculating the average of 12 test results.

### 2.7. Induction Heating Speed and Natural Cooling Speed Test

**Figure 3:**
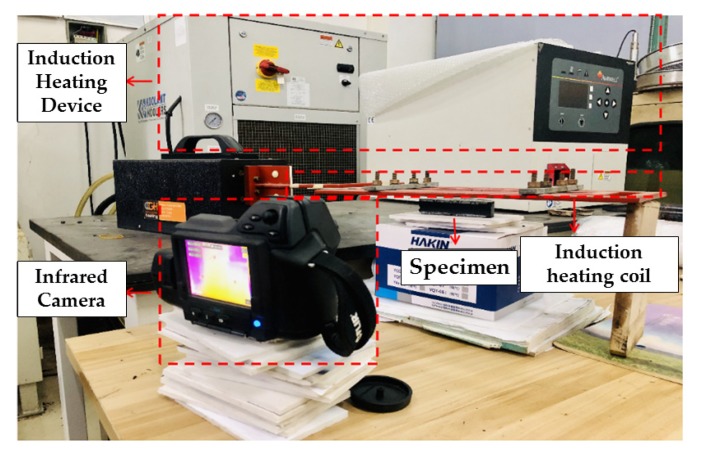
Induction heating test system.

**Figure 4:**
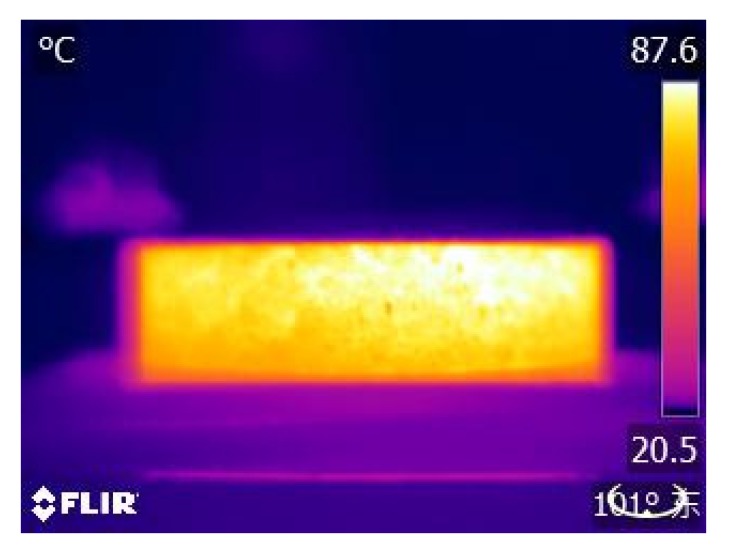
Infrared image of sample exposed to induction heating.

Different steel fiber volumes and different kinds of aggregates can influence induction heating characteristics and cooling properties seriously. Except BA and SS, four other asphalt concretes were heated by an induction heating device with a power of 7.9 kW and a frequency of 123 kHz until the surfaces temperature of the samples reached 90 °C. The surface temperature of the sample was recorded by an infrared camera with pixels of 320 × 240, and the cooling period was also recorded after the surfaces temperature of the samples reach 90 °C. The test system is shown in Figure 3. The sample was placed 10 cm below the induction heating coil. The temperature of the top surface of the sample was measured every 10 s. Figure 4 shows the sample exposed to induction heating.

### 2.8. Induction-Healing Property Test

**Figure 5:**
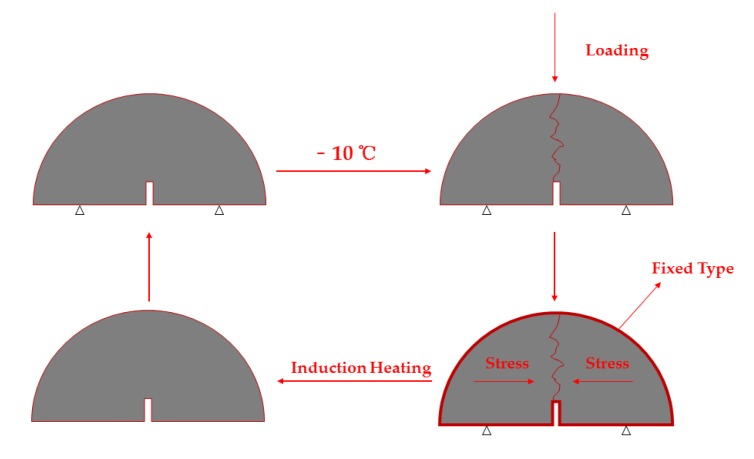
Fatigue-Healing-Fatigue test.

The healing properties were evaluated by the fatigue–healing–fatigue test shown in Figure 5. The specific steps were carried out as follows:(1) First, the semi-circular Marshall test piece was kept in −10 °C for 4 h, and then the sample was subjected to a three-point bending test by UTM-25 to obtain an initial breaking strength *F*_1_ After the test piece was broken, it was placed on a flat surface until it returned to room temperature; (2) The piece was fixed by type for 24 h [34]; (3) Following the same induction heating method as Section 2.7, the test piece was heated to the target temperature. Then, the test pieces were placed in room temperature to cool down; (4) According to step (1), the pieces were broken again to obtain the breaking strength *F*_2_ and then the healing rate was calculated using:

$$R=\frac{F_{2}}{F_{1}}\;\times 100$$

R: Healing Rate, %
*F*_1_: Initial Breaking Strength, kN;
*F*_2_: Terminal Breaking Strength, kN.

**Figure 6:**
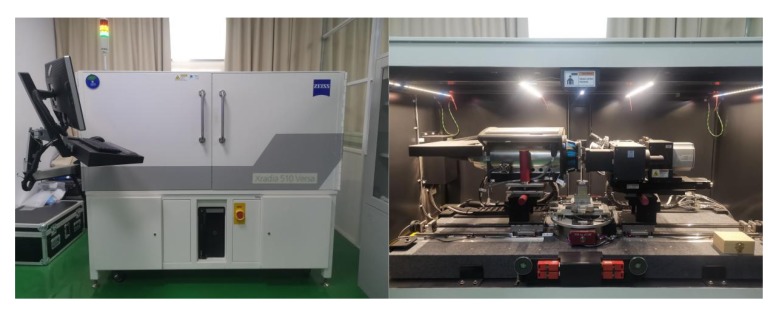
Experimental diagram of the computed tomography (CT) scanning test.

At the same time, a CT scanning (Xradia 510 Versa, ZEISS, Oberkochen, Germany) machine was used to detect the crack dimension of asphalt concrete before and after induction healing in this study. Figure 6 shows the CT scanning machine. The width of the crack is an index to evaluate whether healing is successful. The machine’s actual spatial resolution is 0.7 microns and voxel sizes are as low as 70 nanometers. The temperature in the environmental chamber was 50 °C.

## 3. Results and Discussion

### 3.1. Surface Texture and Pore Sizes Analysis

**Figure 7:**
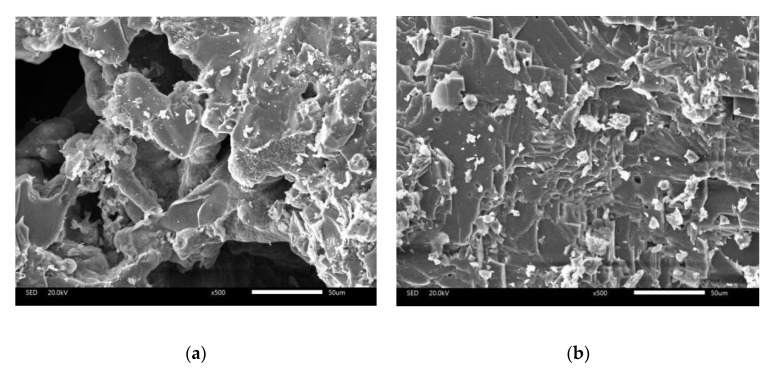
SEM morphology of (**a**) steel slag, (**b**) basalt.

The surface texture of the steel slag and basalt were studied by scanning electron microscopy (SEM). Figure 7 shows the micrographs of steel slag and basalt, respectively. The morphology of steel slag is depicted in Figure 7a. There are many pores on the surface of steel slag while the pores on the surface are considered to be the reason for increasing the amount of asphalt and increasing the surface roughness. Figure 7b shows a plate-like structure and dense surface of basalt.

BJH: a model named after the names of Barret, Joyner, and Halenda. It represents the mesoporous pore size distribution of pores with a width of 2–50 nm.

**Figure 8:**
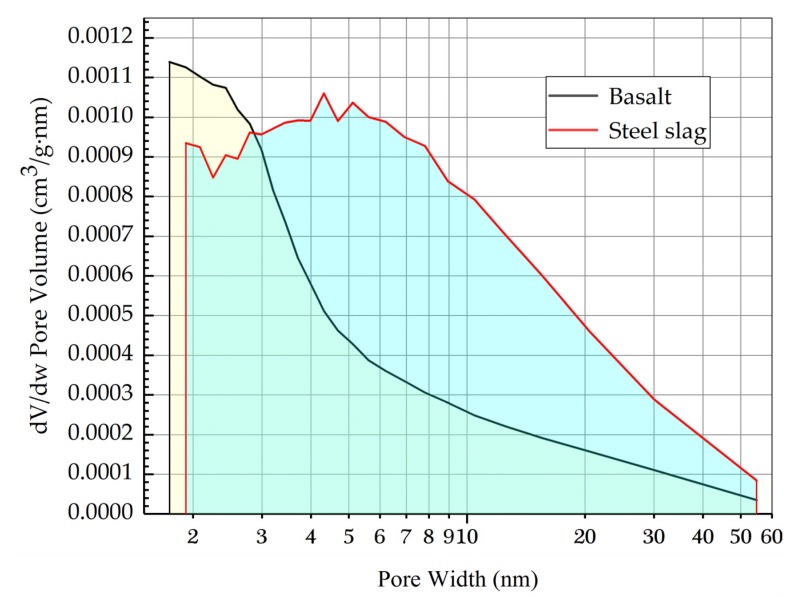
BJH adsorption dV/dw pore volume.

Table 3 shows the difference of surface area between basalt and steel slag. Compared to basalt, steel slag possesses a larger surface area, which for steel slag is nearly 1.67 times that of basalt. In Figure 8, the pore size distribution in steel slag is concentrated at 2~10 nm, while the pore size distribution in basalt is concentrated at 2~3 nm, with 0.008 used cm^3^/g·nm as a standard. After calculations, the area of the shaded portion of steel slag is larger than that of basalt, which indicates that steel slag possesses more pores per volume.

### 3.2. Moisture Susceptibility Analysis

Freeze–thaw splitting is the most severe test to evaluate the water resistance of asphalt concrete. As shown in Table 4, all the asphalt concrete satisfied TSR of 75%. BA-6 possesses higher TSR than BA, which shows the addition of steel fibers increases water resistance. SS, SS-2, SS-4 and SS-6 can draw the same conclusion; the more steel fibers are added, the better water resistance asphalt concrete possesses. BA-6 has higher TSR than SS-6, while BA has higher TSR than SS, which indicates that steel slag has a negative impact on the resistance.

### 3.3. High-Temperature Deformation Resistance

**Figure 9:**
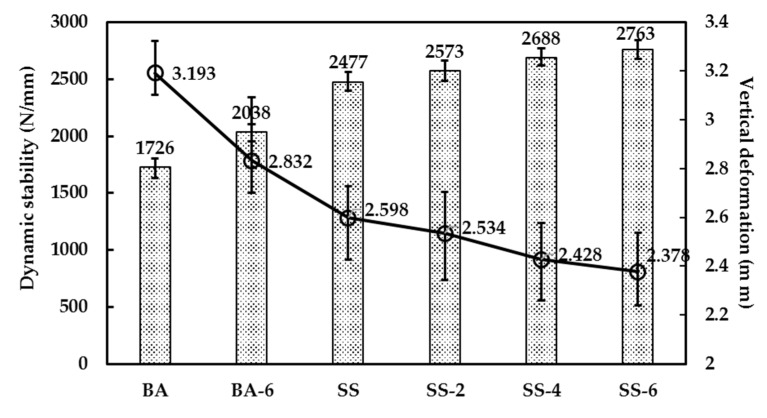
Dynamic stability and vertical deformation of different asphalt concrete.

According to Figure 9, with steel fibers added, the dynamic stability of BA-6 increases by 18% compared to BA, and that of SS-6, SS-4 and SS-2 increases by 12%, 9%, and 4%, respectively, compared to SS. The result shows that the steel fiber is an advantage to high temperature deformation resistance. Replacement of steel slag as an aggregate contributes to increasing dynamic stability by SS-6 increasing by 34% compared to BA-6 and SS increasing by 43% compared to BA. It shows that both steel fibers and steel slag have improved performance on the high temperature deformation resistance of asphalt concrete. The vertical deformation ranking of the six asphalt concretes was: SS-6 > SS-4 > SS-2 > SS > BA-6 > BA, which can help draw similar conclusion to above.

### 3.4. Semi-Circle Bending Fracture Test

**Figure 10:**
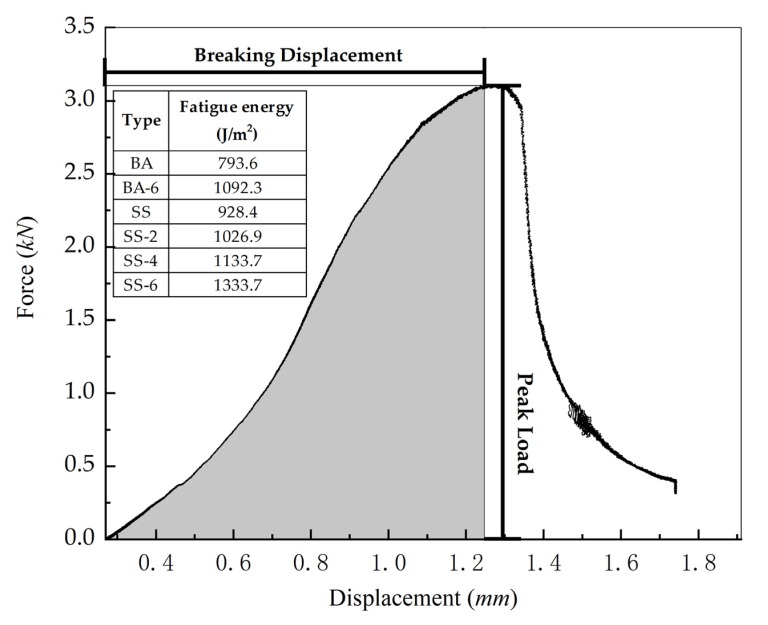
Displacement-Force curve of different asphalt concrete.

**Figure 11:**
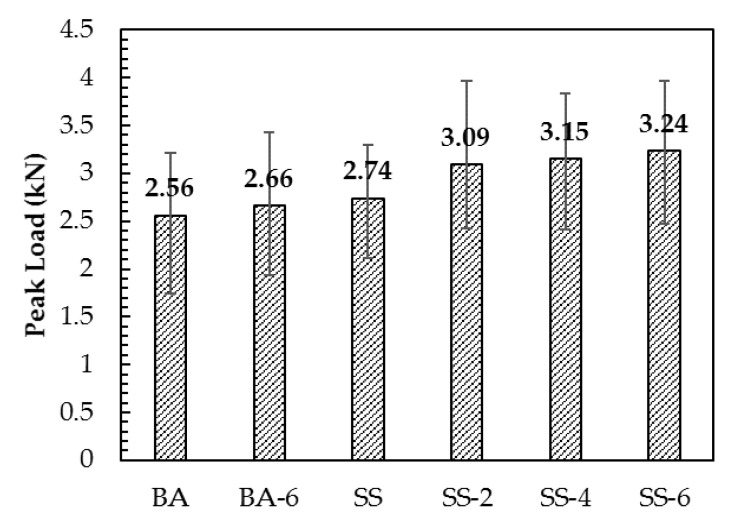
Peak load of different asphalt concretes.

According to Figure 10 and Figure 11, the fracture energy and peak load of the six asphalt concretes were calculated. The fracture energy ranking of the six asphalt concretes was: SS-6 > SS-4 > SS-2 > SS > BA-6 > BA. Compared with SS, the fracture energy of SS-2, SS-4 and SS-6, increased by 10.6%, 22.1%, and 43.7%, respectively, and peak load increased by 12.7, 14.9, and 18.2%. Similar conclusions can be drawn from asphalt concretes with basalts as the aggregates. Compared with BA, BA-6 has an increase in fracture energy of 37.7% and a peak load increase of 3.9%. The results show that the addition of steel fibers increases the fracture energy and peak load of the asphalt concretes. Steel fibers can not only cause the shape of the crack to bend, but increase the damage area, and then consume more energy.

Compared to BA, the fracture energy of SS is increased by 17%, and peak load is increased by 7%. The fracture energy of SS-6 is also improved compared with BA-6. Steel slag can adhere with an asphalt binder outstandingly and has high strength characteristics compared to basalts, which makes the tensile strength of the asphalt concrete with the steel slag as the aggregate larger, increasing the damage energy.

### 3.5. Thermal Properties

The thermal constants of the asphalt concretes are shown in Table 5. Compared with BA, BA-6 has an increase in thermal conductivity of 2.7%, an increase in thermal diffusivity of 7.4%, and a decrease in specific heat capacity of 3.8%. It indicates that steel fibers have the ability to increase the thermal conductivity and thermal diffusion while decreasing the specific heat of asphalt concretes. With the increase of steel fibers content, the thermal conductivity and thermal diffusivity of SS, SS-2, SS-4 and SS-6 gradually increase, while the specific heat capacity gradually decreases. Steel slag influences the thermal constants conversely. Compared with BA, the thermal conductivity and thermal diffusivity of SS were reduced by 38.5% and 55.2%, but the specific heat capacity increased by 10.1%.

The thermal constants of different types of asphalt concretes are different, the main reason of which is that the thermal characteristics of the basic materials are different. Steel fibers are provided with more outstanding conductivity and diffusion of heat properties as a kind of metal material. Although steel slags possess a few metal oxides, there are a lot of pores in steel slags, and the pores have the capacity to set by heat, which blocks heat transfer and diffusion, decreasing the conductivity and thermal diffusion of the mixture but increasing specific heat.

### 3.6. Heating and Cooling Speed

**Figure 12:**
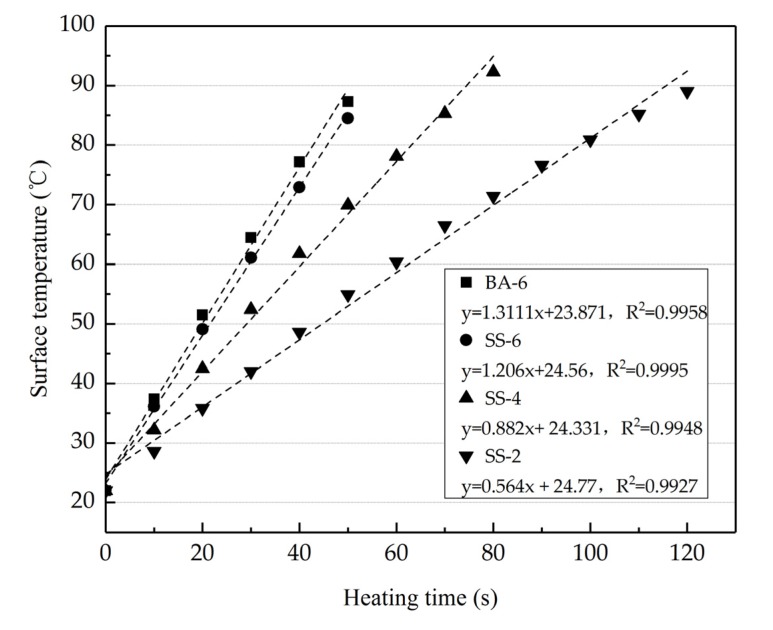
Heating time of different asphalt concrete.

**Figure 13:**
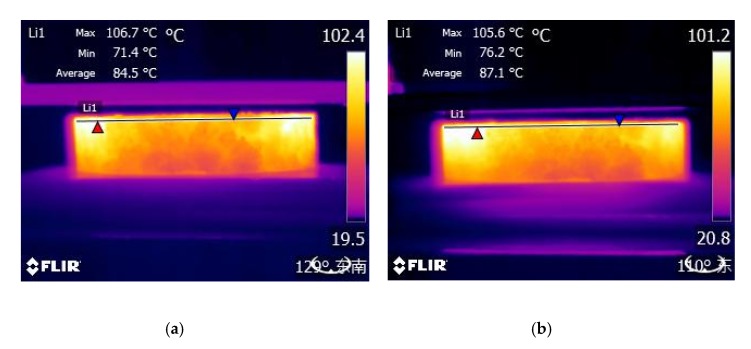
Infrared images of (**a**) SS-6, (**b**) BA-6, exposed during 50 s to induction heating.

Figure 12 shows the surface temperature of the four kinds of asphalt concretes during induction heating and cooling periods. It can be condensed as heating time increases, and the surface temperature increases with line. The slope of the heating line is the heating speed. It can be calculated that the heating speed of BA-6, SS-2, SS-4 and SS-6 were 1.311, 0.564, 0.882, 1.206 respectively according to Figure 12. Steel fibers can be heated quickly and produce a large amount of heating under electromagnetic induction, and it can be concluded that more steel fibers content means quicker heating speed and more heating. Owing to steel slags contains more pores, heating speed of SS-6 is a little lower than BA-6, and the surface temperature of BA-6 is a little higher than SS-6 during same heating time shown in Figure 13. However, it can be found that the max temperature of SS-6 is higher than BA-6, which may be due to that steel slag contains little mental element that can be heated under the influence of a magnetic field [35].

**Figure 14:**
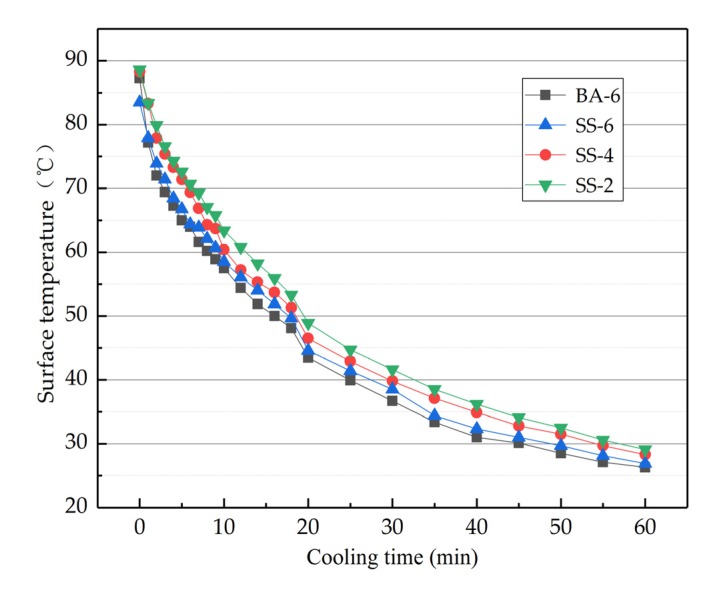
Cooling time of different asphalt concrete.

As shown in the Figure 14, the cooling speed ranking of the four kinds of asphalt concretes was BA-6 > SS-6 > SS-4 > SS-2. Compared to BA-6, SS-6 cooled down more slowly due to the fact that pores make steel slags possess outstanding heat storage. With the content of steel fibers increasing, the asphalt concretes’ cooling speed also grew in number.

### 3.7. Induction-Healing Property

**Figure 15:**
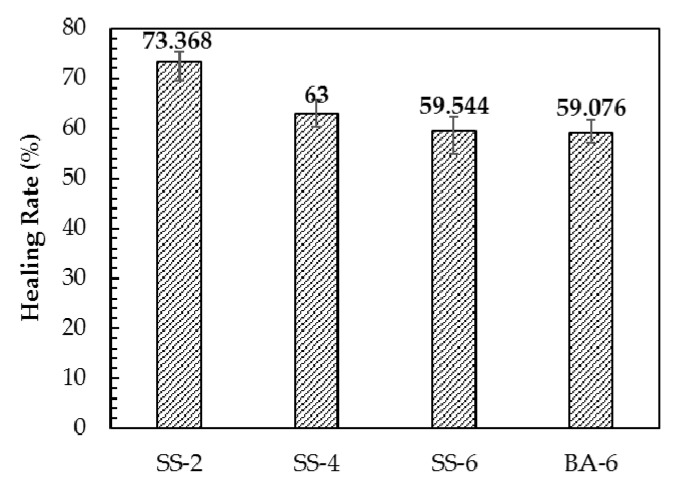
Healing rate of different asphalt concrete.

The healing efficiencies of different types of mixtures are shown in Figure 15. At the optimal healing temperature, the healing efficiency of the four asphalt concretes is ranked as: SS-2 > SS-4 > SS-6 > BA-6. The healing rate of the four asphalt concretes did not exceed 50%. This phenomenon was related to aggregate crushing. The self-healing process can only occur at the mortar, however, once the aggregate breaks, the fracture interface cannot heal. This is an important factor leading to a low rate of healing. It can also be observed from Figure 15 that the rate of healing is inversely related to the steel fiber content. Firstly, this phenomenon is related to the heating time. In order to achieve the same optimum healing temperature, asphalt concrete with a lower steel fiber content needs to be heated for a longer period of time, which results in a longer time for the asphalt to flow and a better healing effect. Secondly, the asphalt concrete with low steel fiber content has a slow cooling rate, which leads to a long time for the asphalt to maintain flow dynamics after the same optimum healing temperature is reached. Compared with BA-6, the healing rate of SS-6 is improved. This is because the larger specific heat capacity of steel slag makes SS-6 cooling slower, so the healing effect is better.

### 3.8. CT Scanning

**Figure 16:**
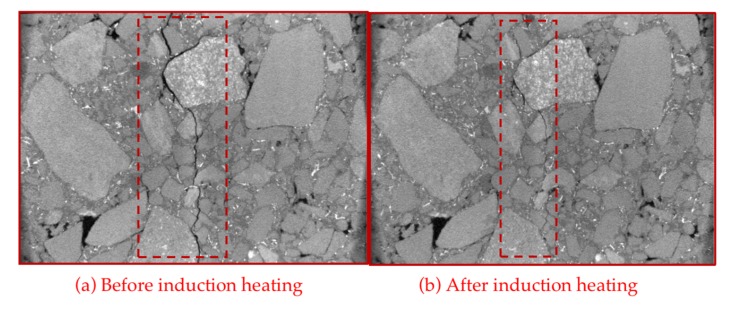
CT scanning figure of SS-6 before and after induction heating.

Figure 16a was scanned immediately after step (1) in Section 2.8, *F*_1_ was obtained and the specie returned to room temperature. After step (3) in Section 2.8, the specie was scanned again, and Figure 16b was obtained. How the healing property acts also depends on the time interval between the scans, so it should be unified for the accuracy of the experiment.

The CT scanning figure of SS-6 before and after induction heating is shown in Figure 15. Before induction heating, the crack in the asphalt concrete is easy to observe in Figure 16a among which there is a crack due to aggregate breaking. After induction heating, the crack shown in Figure 16b is hard to recognize when disregarding the lower part in the figure. At the upper part, there is a little crack more obvious than other cracks. This kind of crack is generated by aggregate breaking, so it is a little harder to be healed than other cracks. However, the width of this kind of crack also can be decreased by asphalt connections. Therefore, it can be concluded that the healing occurs in steel slag induction heating asphalt concrete.

## 4. Conclusions 

In this study, the feasibility that steel slag is applied in induction heating asphalt concretes to replace coarse and fine aggregates is evaluated. Based on the results presented above, the following conclusions can be drawn:

(1) Steel slag contains more pores on the surface while basalt shows plate-like structure and dense surface, and steel slag possesses more pores in volume, which causes the asphalt concrete with basalt aggregate replaced by steel slag holds better heating storage capacity.
(2) Steel fibers contribute to increasing the resistance to moisture damage, high temperature deformation resistance and mechanical performance, while steel slag is beneficial to high temperature deformation resistance, mechanical performance but negative to the resistance to moisture damage due to the presence of f-CaO.
(3) With replacement of steel slag, the asphalt concrete has normal heating time and longer cooling time. More time is provided to heal for the steel slag induction of healing asphalt concretes.
(4) Healing in steel slag induction heating asphalt concrete is successful, and with steel fibers content decreasing, healing of steel slag induction healing asphalt concrete increases. Furthermore, healing is similar between steel slag and basalt induction healing asphalt concretes.
(5) With the same asphalt aggregate ratio, steel slag asphalt concrete shows similar moisture susceptibility, better high-temperature deform resistance, identical induction healing property to basalt asphalt concrete, which means that asphalt concrete using steel slag can work as well as asphalt concrete using basalt without more asphalt added, and the cost of more asphalt can be decreased.
(6) Overall, it is concluded that steel slag can apply in induction heating asphalt concretes to replace coarse and fine aggregates.

## Figures and Tables

**Table 1:** Properties of SBS modified asphalt and basalt aggregate.

Materials	Properties	Values	Specifications	Standard
Asphalt	Penetration (25 °C, 0.1 mm)	68.9	60–80	T0604-2011
	Ductility (15 °C, cm)	>100	≥40	T0605-2011
	Softening point (°C)	49	≥43	T0606-2011
	Density (g/cm^3^)	1.035	-	T0603-2011
Basalt	Fine aggregates density (g/cm^3^)	2.88	≥2.6	T0328-2005
	Coarse aggregates density (g/cm^3^)	2.95	≥2.5	T0304-2005

**Table 2:** Properties of steel fibers and steel slags.

Materials	Properties	Values	Specifications
Steel fibers	Average length (mm)	4.2	-
	Equivalent diameter (μm)	70–130	-
	Density (g/cm^3^)	7.8	-
Steel slag	Fine aggregates density (g/cm^3^)	3.56	≥2.9
	Coarse aggregates density (g/cm^3^)	3.65	≥2.9
	Los Angeles abrasion	8.3	≤28
	Crush values	12.9	≤26

**Table 3:** Surface area of basalt and steel slag.

Aggregate	Single Point Surface Area at P/Po (m^2^/g)	BET Surface Area (m^2^/g)
Basalt	6.0915	6.3051
Steel slag	10.1707	10.5559

**Table 4:** Result of freeze–thaw splitting of different asphalt concretes.

Type	R_T1_ (MPa)	R_T2_ (MPa)	TSR (%)
BA	1.121	0.963	85.9
BA-6	1.203	1.082	89.9
SS	1.249	1.022	81.8
SS-2	1.293	1.077	83.3
SS-4	1.343	1.138	84.7
SS-6	1.421	1.225	86.2

R_T1_: Average of splitting tensile strength of specimens without freeze–thaw cycles; R_T2_: Average of splitting tensile strength of specimens after freeze–thaw cycles; TSR: Intensity ratio of the freeze–thaw split test, TSR = (R_T1_/R_T2_) × 100.

**Table 5:** Thermal properties of different asphalt concrete.

Mixture Type	Thermal Conductivity (W/mK)	Thermal Diffusivity (mm^2^/s)	Specific Heat (MJ/m^3^K)
BA	1.08	1.35	0.80
BA-6	1.11	1.45	0.77
SS	0.78	0.87	0.89
SS-2	0.86	0.89	0.97
SS-4	0.91	0.97	0.94
SS-6	0.94	1.04	0.91
